# Mitochondrial ageing and antiretroviral therapy exposure

**DOI:** 10.1186/1758-2652-13-S4-O28

**Published:** 2010-11-08

**Authors:** BAI Payne, CA Hateley, DC Samuels, IJ Wilson, M Santibanez-Koref, DA Price, PF Chinnery

**Affiliations:** 1 Newcastle University, Institute of Human Genetics, Newcastle-upon-Tyne, UK; 2 Newcastle University, Institute for Ageing and Health, Newcastle-upon-Tyne, UK; 3 Vanderbilt University, Centre for Human Genetics Research, Nashville, TN, USA; 4 Newcastle University, Mitochondrial Research Group, Newcastle-upon-Tyne, UK; 5 Royal Victoria Infirmary, Department of Infection & Tropical Medicine, Newcastle-upon-Tyne, UK

## Purpose

Normal human ageing is thought to be driven by the progressive accumulation of molecular defects, including somatic mitochondrial DNA (mtDNA) mutations. Certain nucleoside analogue anti-retroviral drugs (NRTIs) are known to cause reversible mtDNA depletion, but it is unknown whether they affect age-associated mtDNA mutation.

## Methods

We have recruited adult HIV-infected patients, all aged 50 years or under. Subjects were stratified according to cumulative (lifetime) exposure to those NRTIs previously implicated in disruption of mtDNA replication. Proportional level of the age-associated mtDNA common deletion (CD) was measured by means of a novel real-time PCR assay. Based on these and prior observations from our group, we then developed a validated model of mtDNA replication [1,2], and incorporated a period of partial mtDNA replication failure due to NRTI exposure.

## Results

Amongst all patients CD levels increased with subject age (r=0.467, p=0.005). Mean CD levels were significantly higher in NRTI-exposed than unexposed patients (mean log_10_(CD/mtDNA) ±SEM: NRTI+, -3.46 ±0.24? NRTI-, -4.62 ±0.29? p=0.006). Lifetime NRTI exposure was predictive of CD level (r=0.419, p=0.052). In silico modelling demonstrated that a finite period of partial replication failure was seen to lead to a period of mtDNA depletion during the exposure which corresponded to that expected for the relevant NRTI. During this period rapid expansion of pre-existing mtDNA deletion mutations was observed within individual simulated cells. This effect led to an increase in the proportion of cells with a functional mitochondrial defect which continued to increase after the period of exposure due to the continued effects of ageing. Longer exposure, exposure to more potent inhibitors of mtDNA replication and exposure later in life had the most profound effects on eventual cellular defect.

## Conclusions

Cumulative exposure to certain NRTIs accelerates the accumulation of age-associated mtDNA deletion mutations, which appears to be irreversible, and mirrors that expected much later in life due to normal ageing. This effect could be caused simply by accelerated expansion of pre-existing age-associated somatic mtDNA mutations, mediated by finite periods of NRTI exposure. These data plausibly provide a novel biological mechanism for the phenomenon of accelerated ageing recently described in long-term treated HIV-infected patients.
